# TRIOBP modulates β-catenin signaling by regulation of miR-29b in idiopathic pulmonary fibrosis

**DOI:** 10.1007/s00018-023-05080-4

**Published:** 2023-12-29

**Authors:** Lan Wang, Wenyu Zhao, Cong Xia, Shuaichen Ma, Zhongzheng Li, Ningdan Wang, Linke Ding, Yaxuan Wang, Lianhui Cheng, Huibing Liu, Juntang Yang, Yajun Li, Ivan Rosas, Guoying Yu

**Affiliations:** 1 State Key Laboratory of Cell Differentiation and Regulation; Henan International Joint Laboratory of Pulmonary Fibrosis; Henan Center for Outstanding Overseas Scientists of Organ Fibrosis; College of Life Science, Henan Normal University, 46 Jianshe Road, Xinxiang, 453007 Henan China; 2 Division of Pulmonary, Critical Care and Sleep Medicine, Baylor College of Medicine, Houston, TX 77030 USA

**Keywords:** Anti-fibrotic axis, Fibroblast activation, Epithelial–mesenchymal crosstalk

## Abstract

Idiopathic pulmonary fibrosis (IPF) is a fatal and devastating lung disease of unknown etiology, described as the result of multiple cycles of epithelial cell injury and fibroblast activation. Despite this impressive increase in understanding, a therapy that reverses this form of fibrosis remains elusive. In our previous study, we found that miR-29b has a therapeutic effect on pulmonary fibrosis. However, its anti-fibrotic mechanism is not yet clear. Recently, our study identified that F-Actin Binding Protein (TRIOBP) is one of the target genes of miR-29b and found that deficiency of TRIOBP increases resistance to lung fibrosis in vivo. TRIOBP knockdown inhibited the proliferation of epithelial cells and attenuated the activation of fibroblasts. In addition, deficiency of Trio Rho Guanine Nucleotide Exchange Factor (TRIO) in epithelial cells and fibroblasts decreases susceptibility to lung fibrosis. TRIOBP interacting with TRIO promoted abnormal epithelial–mesenchymal crosstalk and modulated the nucleocytoplasmic translocation of β-catenin. We concluded that the miR-29b‒TRIOBP–TRIO–β-catenin axis might be a key anti-fibrotic axis in IPF to regulate lung regeneration and fibrosis, which may provide a promising treatment strategy for lung fibrosis.

**Graphical Abstract:**

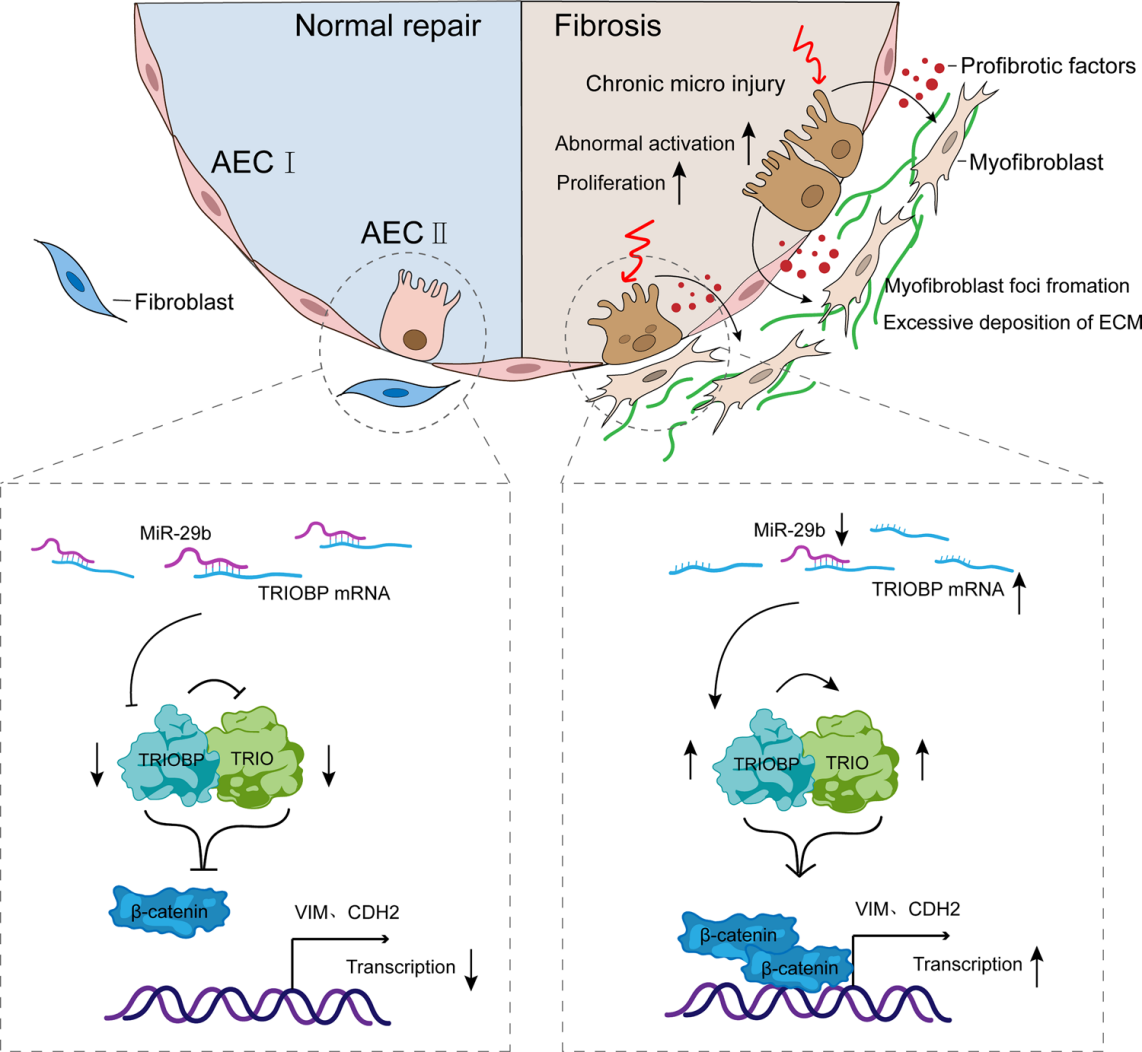

**Supplementary Information:**

The online version contains supplementary material available at 10.1007/s00018-023-05080-4.

## Introduction

Idiopathic pulmonary fibrosis (IPF) is a fatal and devastating lung disease of unknown etiology [1]. Several risk factors, including aging, smoking, gastroesophageal reflux, and environmental variables, have been proposed associated with IPF [2–4]. In IPF conditions, this becomes a progressive and unstoppable process, resulting in severe lung scarring and respiratory failure. Although it has been challenging to pinpoint the pathogenic factors causing IPF, lung epithelial cells are likely to play a major role in IPF [5, 6]. Injuries to lung epithelial cells caused by a variety of insults are thought to be the first step in the pathogenesis of pulmonary fibrosis. This is followed by basement membrane (BM) disruption, epithelial cell death and the epithelial–mesenchymal transition (EMT), infiltration of inflammatory cells, fibroblasts differentiation into active myofibroblasts, extracellular matrix (ECM) overproduction and deposition, and scar formation. Alveolar epithelial type II cells (AECIIs) can serve as progenitor cells that preserve the homeostasis of the epithelium and restore damaged epithelium following injury [7–9]. Activated alveolar epithelial cells could release a variety of cytokines and profibrogenic growth factors, such as transforming growth factor β (TGF β), resulting in aberrant epithelial–mesenchymal crosstalk and recruitment of highly synthetic and contractile myofibroblasts, with deposition and remodeling of the ECM.

MicroRNAs (MiRNAs) are deeply involved in regulating processes implicated in IPF such as lung development [10–13] and maintenance of cellular phenotypes [10, 11, 14–16]. Furthermore, IPF lungs exhibit a significantly altered miRNA repertoire [17, 18]. Among miRNAs, the microRNA-29 (miR-29) family is well characterized for their ability to regulate extracellular matrix proteins and has been extensively studied as a potential anti-fibrotic regulator [19]. MiR-29 is decreased in kidney, lung, liver, and myocardial fibrosis and it is important to fibrosis molecules, such as collagen I and III, insulin-like-growth factor-1 (IGF-1), and connective tissue growth factor (CTGF) [17, 20–27]. Consistent with this finding, using a miR-29 mimics developed by miRagen that exhibits preferential lung distribution when administered systemically, which were able to blunt bleomycin (BLM)-induced pulmonary fibrosis in mice [28, 29], we observed that patients with low serum levels of miR-29 experienced significantly shortened survival times. Supplementing miR-29 could be a therapeutic strategy for reversing or mitigating organ fibrosis. With specific relevance to IPF, expression of members of the miR-29 family is decreased in the human IPF lung as well as in animal models of lung fibrosis and their targets are increased. Our previous studies have revealed that miR-29b was downregulated in IPF and BLM-induced lung fibrosis; in addition, treatment with miR-29b mimic in the context of pulmonary fibrosis significant preserves the BLM-induced decrease of miR-29b and reverses pulmonary fibrosis [29]. Several previous studies also reported that miR-29 has been verified to act as a vital regulator in various diseases [30].

Trio Rho Guanine Nucleotide Exchange Factor (TRIO) can encode a large protein that functions as a GDP to GTP exchange factor, which promotes the reorganization of the actin cytoskeleton [31]. Several studies have linked TRIO with intellectual disability [32] and autism spectrum disorders [33–35]. One report implicated that the absence of TRIO results in embryonic mortality as well as defective skeletal muscle development [36]. TRIO-mediated RhoA activation is important during early eye development [37] and TRIO-regulated MYH9 activation is essential for craniofacial abnormalities in zebrafish [38]. Recently, studies showed that TRIO regulates cell migration, growth, or invasion, thereby promoting the development of cervical cancer [39], osteosarcoma [40], breast cancer [41], colorectal cancer [42], and liver cancer [43]. TRIO and F-actin-binding protein (TRIOBP) also referred to as Tara, was originally isolated as a cytoskeleton remodeling protein [44]. Recent studies provide clues that TRIOBP variants are associated with other human diseases including cancer and brain diseases. However, TRIOBP and TRIO exact function in IPF as well as the underlying mechanism are still largely unknown. In our research, we demonstrate that TRIOBP was one of target genes of miR-29b. TRIOBP interacts with TRIO and positively regulates TRIO expression. TRIO is upregulated in IPF lung tissues and experimental pulmonary fibrosis. TRIO knockdown significantly reduced the proliferation, migration, and EMT process of AECIIs, as well as inhibits lung fibroblast activation. TRIO knockdown specifically inhibited the nuclear deposition of β-catenin, thereby inhibited some profibrotic genes expression. TRIO and TRIOBP play a stimulative role in aberrant epithelial–mesenchymal crosstalk, which might drive the progress of IPF. Our study not only expands on the understanding of the mechanism for IPF, but also provides a theoretical basis for the research and development of new target for pulmonary fibrosis.

## Materials and methods

### Lung tissue sampling

Lung tissue samples were obtained from the Henan Provincial Chest Hospital. The IPF samples were surgical remnants of biopsies or lungs explanted from patients with IPF undergoing pulmonary transplant. Control was normal histology tissue obtained from normal disease-free margin of lung cancer resection specimens. IPF was diagnosed based on ATS/ERS/JRS/ALAT Clinical Practice Guidelines [45]. The clinical characteristics of all patients are summarized in Supporting Information Table S1. All studies were approved by the Henan Provincial Chest Hospital Medical Research Ethics Committee (No.2020-03-06). The research conformed to the principles of the Declaration of Helsinki. Oral and written informed consent was obtained from all patients.

### Generation of mouse model with lung fibrosis

All animal procedures were approved and performed in accordance with the guidelines of the Institutional Animal Care and Use Committee at the Henan Normal University (IACUC, SMKX-2118BS1018). C57BL/6 mice (5–6 weeks old) were randomly divided into four groups: a saline group, a BLM-induced pulmonary fibrotic group, AAV-shTriobp + saline group, and AAV-shTriobp + BLM group. C57BL/6 mice were allowed to acclimate for 1 week prior to intratracheal administration of either 2.5 × 10^11^ µg of AAV-shTriobp or AAV-NC (OBiO Technology Corp., Ltd.). After 7 days, pulmonary fibrosis was induced by tracheal infusion of bleomycin (1.5 U/kg), which was carried out under light anesthesia (3–4% isoflurane). ShTriobp: GACGGATTCAAGCCTCAAATA. 14 days after BLM injection, for tissue collection, mice were given a lethal dose of urethane intraperitoneally.

### Hydroxyproline assay

Collagen deposition was determined by measuring the total hydroxyproline content in wet lung tissue, which was measured by a hydroxyproline assay kit (Nanjing Jiancheng Bioengineering Institute, AO303-1), according to the manufacturer’s protocol. The concentration of right lung hydroxyproline (µg/mg of wet tissue) was calculated.

### Cell culture and treatment

A549, MRC5, 293 T, and MLE-12 cells were obtained from American Type Culture Collection. A549, MRC5, MLE-12, primary mouse lung fibroblast (PMLFs), and 293 T cells were cultured in DME/F-12 or DMEM at 37 °C and 5% CO_2_. The medium contained 10% fetal bovine serum (FBS), 100 units/mL penicillin, 100 g/mL streptomycin, and 1 mM sodium pyruvate. Media were changed every 3 days.

Mimic miR-29b was synthesized at miRagen Therapeutics, Inc. utilizing standard phosphoramidite solid phase synthesis. Inhibitor miR-29b was obtained from Sangon Biotech (Shanghai) Co., Ltd. The control duplex sequence does not target any known murine or human transcripts by BLAST analysis. The transfection reagent used to mimic miR-29b and inhibitor miR-29b was obtained from Guangzhou RiboBio Co., Ltd.

### Isolation of PMLFs

In brief, mice were euthanized with anesthetics and cleaned with 75% ethanol. The lung tissue was collected and washed twice with sterile PBS before being cut into 1 mm^3^ pieces. The pellet was resuspended in DMEM medium containing 15% FBS and cultured in 10-cm culture dishes at 37 °C after centrifugation (600 g, 5 min). The attached fibroblasts were collected after 4–5 days of incubation for sub-culturing or other assays.

### Hematoxylin and eosin (H&E) and Masson’s trichrome staining

H&E and Masson’s trichrome staining were performed according to the instructions of the kit (Solarbio, Beijing, China), and histopathological changes were observed under a microscope.

### Immunohistochemistry (IHC)

IHC was performed on TRIOBP (Abcam, ab151320) and TRIO (Affinity, DF2685) expression in IPF and BLM-treated mouse lung. The steps of IHC were as follows: lung sections dewaxed transparently and treated with 3% H_2_O_2_ dropwise for 10 min; washed with distilled water and immersed the slides in 0.01% sodium citrate solution, microwave to boiling, 50% goat serum blocked 30 min, overnight incubation with primary antibody. The following day, the horseradish peroxidase-labeled antibody was incubated for 30 min at 37 °C before developing with DAB chromogen. After staining the nuclei with hematoxylin for 5 min, the sections were rinsed with running water for 10 min. Five visual fields were randomly selected for evaluation in each section after being sealed with neutral balsam.

### Quantitative real-time PCR

Total RNA was isolated using TRIzol reagent. cDNA was synthesized by M-MLV Reverse Transcriptase (Promega, M1708). RT-qPCR was conducted using SYBR green kit (Yeasen, 11201ES03) according to the manufacturer’s instructions. Each sample and experiments were tested in triplicate. Delta CT values of target gene were normalized to *GAPDH* or *ACTB*. The data were evaluated by the 2^−ΔΔCt^ method. RT-qPCR primers used are as described in Table 1.

**Table 1:** Primers used in the study (RT-qPCR)

Gene	Primer	Sequence 5′ > 3′
TRIO	Sense	AGGCCGAAAAGTATATGAGCAAC
	Anti-sense	GTCAAGGAGCGACTTCCCAT
TRIOBP	Sense	TCCAAGGTCTCCCTTAGTACA
	Anti-sense	GTGGGACTGGACTTGCTA
ACTA2	Sense	CTCTGGACGCACAACTGGCATC
	Anti-sense	CACGCTCAGCAGTAGTAACGAAGG
COL1A1	Sense	GAGGGCCAAGACGAAGACATC
	Anti-sense	CAGATCACGTCATCGCACAAC
FN1	Sense	ACAACACCGAGGTGACTGAGAC
	Anti-sense	GGACACAACGATGCTTCCTGAG
VIM	Sense	TTGCCGTTGAAGCTGCTAACTACC
	Anti-sense	AATCCTGCTCTCCTCGCCTTCC
ACTB	Sense	CACCATTGGCAATGAGCGGTTC
	Anti-sense	AGGTCTTTGCGGATGTCCACGT
GAPDH	Sense	GTCTCCTCTGACTTCAACAGCG
	Anti-sense	ACCACCCTGTTGCTGTAGCCAA

### Western blot analysis and antibodies

Proteins were extracted from cell lysates in lysis buffer and used to quantify protein levels. Proteins were separated on polyacrylamide gel electrophoresis, transferred to polyvinylidene difluoride membranes, and immunoprobed with specific antibodies. The proteins were detected using a chemiluminescence reagent kit purchased from Thermo Fisher Scientific. The imager station captured the images (Odyssey Software Version 5.2, LI-COR Biosciences). Anti-TRIO (Affinity, DF2685), anti-TRIOBP (Proteintech, 16124-1-AP), anti-β-actin (Affinity, AF7018), and anti-GAPDH (Affinity, AF7021) antibodies were purchased from Affinity Biosciences LTD. Anti-α-SMA antibodies were purchased from Abcam (Abcam, ab124964). Anti-vimentin (Proteintech, 10366-1-AP), anti-E-cadherin (Cell Signaling Technology, 14472S), anti-type I collagen (Proteintech, 14695-1-AP) and anti-fibronectin (Proteintech, 15613-1-AP) antibodies were purchased from Proteintech Group.

### Analysis of cell survival and migration

The CCK8 (APExBLO company) and Cell-Light EdU DNA cell proliferation kit (RiboBio, Guangzhou, China) were used to detect the ability of cell proliferation following each manufacturer’s protocol. Cells were plated and grown into a confluent monolayer in six-well plates to test cell migration. After that, scratches were made with a pipette tip. A microscope was used to observe the cell migration process, the following wounding at 0 and 24 or 48 h. Transwell assay was also used to analyze cell migration. Serum-free DME/F12 was used to resuspend the cells, and DME/F12 containing FBS was used in the lower chambers (10%). Calculating the cell numbers from five random fields allowed for the staining, imaging, and analysis of the migrated cells.

### Annexin V-FITC/PI flow cytometry

The experiment was carried out with the Apoptosis Detection Kit (Solarbio, CA1020). A549 cells were digested with trypsin without EDTA (Invitrogen), and centrifuged at 1000 rpm for 5 min to be collected. 5 µL of Annexin V-FITC dye was added into the binding buffer (200 µL). Then they were mixed well, and reacted in room temperature for 15 min away from light. Subsequently, 5 µL PI dyes were added. Similarly, they were mixed well and reacted. Flow cytometry was performed within 1 h using FACSCalibur Flow Cytometer (BD Biosciences, San Jose, CA).

### EdU incorporation assay

An Apollo567 in vitro Imaging Kit was purchased from RiboBio Corporation (Guangzhou, China) and was applied for the EdU incorporation assay. After culturing with EdU (10 μM) for 2 h, the cells were fixed with paraformaldehyde (4%), permeabilized with Triton X-100 (0.2%), and contained with 4′,6-diamidino-2-phenylindole (DAPI, 5 μg/mL) and Apollo fluorescent dyes.

### Immunofluorescence assay

Cells fixed with 4% paraformaldehyde were washed three times for 5 min, blocked with 1% goat serum for 1 h at room temperature (RT), incubated with the required antibody, washed three times in PBS, incubated with secondary antibody, washed three times in PBS again, treated with DAPI to dye the nuclei, and finally washed three times in PBS.

### Plasmid construction, lentivirus package, and stable-infected cell lines construction

Silencing TRIOBP was achieved by targeting the sequences “GCTGACAGATTCAAGTCTCAA” in the pLKO.1 vector; silencing TRIO was achieved by targeting the sequences “CCACGAAGAATGGATTGAAAT” in the pLKO.1 vector; silencing Triobp was achieved by targeting the sequences “GACGGATTCAAGCCTCAAATA” in the pLKO.1 vector; silencing Trio was achieved by targeting the sequences “TCGACCTATCCGTAGCATTAA” in the pLKO.1 vector. These sequences were acquired from sigmaaldrich.cn. Sub-confluent cultures were overnight infected with concentrated lentivirus particles in the presence of 5 g/mL polybrene. In media containing 2 g/mL puromycin, cells were chosen 24 h after transfection. Details for primers are provided in Table 2.

**Table 2:** Primers used in the study

Gene	Primer	Sequence 5ʹ–3ʹ
sh-TRIO	Sense	CCGGCCACGAAGAATGGATTGAAATCTCGAG
		ATTTCAATCCATTCTTCGTGGTTTTTG
	Anti-sense	AATTCAAAAACCACGAAGAATGGATTGAAAT
		CTCGAGATTTCAATCCATTCTTCGTGG
sh-TRIOBP	Sense	CCGGGCTGACAGATTCAAGTCTCAACTCGAG
		TTGAGACTTGAATCTGTCAGCTTTTTG
	Anti-sense	AATTCAAAAAGCTGACAGATTCAAGTCTCAA
		CTCGAGTTGAGACTTGAATCTGTCAGC
sh-Trio	Sense	CCGGTCGACCTATCCGTAGCATTAACTCGAG
		TTAATGCTACGGATAGGTCGATTTTTG
	Anti-sense	AATTCAAAAATCGACCTATCCGTAGCATTAA
		CTCGAGTTAATGCTACGGATAGGTCGA
sh-Triobp	Sense	CCGGGACGGATTCAAGCCTCAAATACTCGAG
		TATTTGAGGCTTGAATCCGTCTTTTTG
	Anti-sense	AATTCAAAAAGACGGATTCAAGCCTCAAATA
		CTCGAGTATTTGAGGCTTGAATCCGTC

### Isolation of conditioned medium (CM)

The medium was replaced and cells were incubated with fresh medium for 72 h. CM was collected, centrifuged, and immediately used for recipient cells incubation (72 h) or stored at − 20 °C for later use.

### ChIP-qPCR assay

ChIP–qPCR was performed as described [39]. A549 cells were cross-linked with 1% formaldehyde for 10 min at RT. Fixation was stopped by 125 mM glycine for 10 min and the samples were washed twice with ice-cold PBS. Cell pellets were then resuspended in 1 mL of cytolysis buffer, mixed and incubated on ice for 10–15 min with occasional inversion every 2 min. Cells were then centrifuged for 5 min at 3500 rpm at 4 °C, the supernatant was discarded, and the remaining nuclear pellet (white) was resuspended in 500 μL of nuclear lysis buffer (1% SDS, 10 mM EDTA, 50 mM Tris–Cl, pH 8.1, 1 × protease inhibitor cocktail). The average length of the shared chromatin is about 250 bp or less. The soluble chromatin was collected by centrifuging for 10 min at 14,000 rpm, and the supernatant was diluted 1:10 with dilution buffer (150 mM NaCl, 20 mM Tris–HCl, pH 8.1, 2 mM EDTA, 1% Triton X-100 and 1 × protease inhibitor cocktail). Chromatin was incubated at 4 °C overnight with protein A/G beads. Anti-rabbit IgG was used as a negative control. The precipitated complexes were washed twice in low-salt buffer (150 mM NaCl, 20 mM Tris–HCl, pH 8.1, 2 mM EDTA, 1% Triton X-100, 0.1% SDS), twice in high-salt buffer (500 mM NaCl, 20 mM Tris–HCl, pH 8.1, 2 mM EDTA, 1% Triton X-100, 0.1% SDS), twice in LiCl buffer (250 mM LiCl, 1% NP-40, 1% deoxycholate, 1 mM EDTA, pH 8.0, 10 mM Tris–HCl, pH 8.1), and twice in TE buffer (pH 8.0). The thoroughly washed beads were eluted twice with 150 μL of elution buffer (0.1 M NaHCO_3_, 1% SDS) by vortexing at 70 °C at 1000 rpm for 10 min on a Thermo Mixer C (Eppendorf). The elutes were pooled and heated at 65℃ for overnight to reverse the formaldehyde cross-link. The enriched DNA fragments were then purified with QIAquick Spin column and quantified by Nanodrop. The published primers of *VIM* and *CDH2* were used for β-catenin occupancy analysis with SYBR green master mix (Yeasen, 11201ES03). The following antibodies were used: anti-β-catenin (proteintech, 51067-2-AP) and anti-IgG (Beyotime, A7016). Primers used for ChIP–qPCR are listed in Table 3.

**Table 3:** ChIP-qPCR primers in the study

Gene	Primer	Sequence 5ʹ–3ʹ
VIM-UP-1	Sense	AACTTAGGGGCGCTCTTGTC
	Anti-sense	GGTGGGGTCGCTTAGTCAC
VIM-UP-2	Sense	AAGCTGGACTGAGCCCGTTA
	Anti-sense	AAAGAGCGCCTGAGATTGGA
VIM-Down-1	Sense	GCTTCGCCAACTACATCGAC
	Anti-sense	CATGGGCGCAGCCTTACTT
VIM-Down-2	Sense	GAGAAGTAAGGCTGCGCCCAT
	Anti-sense	GAGGAAATGCGAACTGCAAGG
CDH2-UP-1	Sense	TTGGCCTGCGTCCTTAGTTT
	Anti-sense	AGGGGCTGCGGGAAATAAAA
CDH2-UP-2	Sense	CGCTCCATTCCACAAATGCTT
	Anti-sense	TTGGGGCCAACAGTTTCAGG
CDH2-Down-1	Sense	CCGGAGAACAGTCTCCAACT
	Anti-sense	GTTTCGGGCTCGTGGTTTTG
CDH2-Down-2	Sense	GAGAACAGTCTCCAACTCGC
	Anti-sense	GGTTTTGCTTCCTCCGGGTC

### Dual-luciferase reporter analysis

Whether there was a direct target between miR-29b and TRIOBP was confirmed by luciferase reporter gene assay. The potential TRIOBP 3ʹUTR’s targets of miR‐29b were acquired from TargetScan (http://www.targetscan.org/vert_72/). The primers of the predicted target gene TRIOBP were from the Sangon Biotech (Shanghai) Co., Ltd. To obtain the sequence of deletion‐type TRIOBP, we use TRIOBP-3ʹUTR-1 and TRIOBP-3ʹUTR-2 primers to construct two short sequences and then let these two sequences use each as a template for another PCR to acquire the deletion‐type TRIOBP. Primer sequences are listed in Table 4. Sequences of wild‐type TRIOBP and deletion‐type TRIOBP were inserted into the pGL3.0 luciferase reporter vector. Ligase 4 was used to ligate the target gene and the TRIOBP‐wild‐type‐Luc and TRIOBP‐deletion‐type‐Luc plasmid vectors. A total of 20 µL plasmid and 100 µL competent cells containing DH5α were then added into 1.5 mL centrifuge tubes, left on ice for 30 min, water‐bathed at 42℃ for heat shock for 90 s, and immediately chilled on ice for 2 min. After this, 37 ℃ pre‐heated 800 µL Luria–Bertani (LB) liquid medium was added to the tube and centrifuged at 37 ℃ at 220 rpm for 45 min. A total of 150 µL DH5α liquid was tiled on the Amp (+) LB plate, which was reversely placed and incubated overnight at 37 ℃. The 293 T, A549, and MRC5 cells were cultured in 24-well plates and transfected with 400 ng luciferase reporter plasmids together with 50 nM miR-29b mimic/inhibitor/ negative control (NC). The empty was only transferred pGL3.0 vector with no insertion sequence. At 36 h after transfection, the Dual-Luciferase Reporter Assay System purchased from Yeasen Biotechnology (Shanghai) Co., Ltd (11402ES80) was used to detect luciferase activity.

**Table 4:** Primers used in the study

Gene	Primer	Sequence 5ʹ–3ʹ
TRIOBP-3ʹUTR	Sense	ACTGGGAGATGGGATGCCTGCC
	Anti-sense	GCTGGTCAGTTTCCTGCCGTGG
TRIOBP-3ʹUTR-1	Sense	ACTGGGAGATGGGATGCCTGCC
	Anti-sense	CAGAGGCTGGCTTGTAGCTGCCCTAGGCAGC
TRIOBP-3ʹUTR-2	Sense	TAGGGCAGCTACAAGCCAGCCTCTGAAAGGTGCTCCAC
	Anti-sense	GCTGGTCAGTTTCCTGCCGTGG

### Lung single cell RNA-seq data analysis from human and mouse

The human single-cell data used in this study are sourced from the Human Cell Atlas, specifically focusing on the cell types present in the human lung. The identification of cell identities is based on the research conducted by Sikkema et al. [46]. Mouse single-cell data are obtained from the work previously published by Wang et al. [47]. The human single-cell data are analyzed using Scanpy [48], a powerful Python library designed for single-cell RNA-seq analysis, with UMAP plots being generated to visualize the cellular landscape. Similarly, Seurat [49], a popular R toolkit for single-cell genomics analysis, is employed for the analysis of the mouse single-cell data.

### Statistical analysis

Every experiment was repeated at least three times. The results in the control and experimental groups were analyzed by GraphPad software 9.0. The Shapiro–Wilk normality test was used to test normal distribution. The results were analyzed by the Mann–Whitney U test for comparisons between two groups when sample data were not normally distributed, and by unpaired Student’s *t* test for comparisons between two groups with normal distribution. Data are presented as mean ± SD and were considered statistically significant at *P* < 0.05.

## Results

### TRIOBP is a directly target gene of miR-29b

**Fig. 1:**
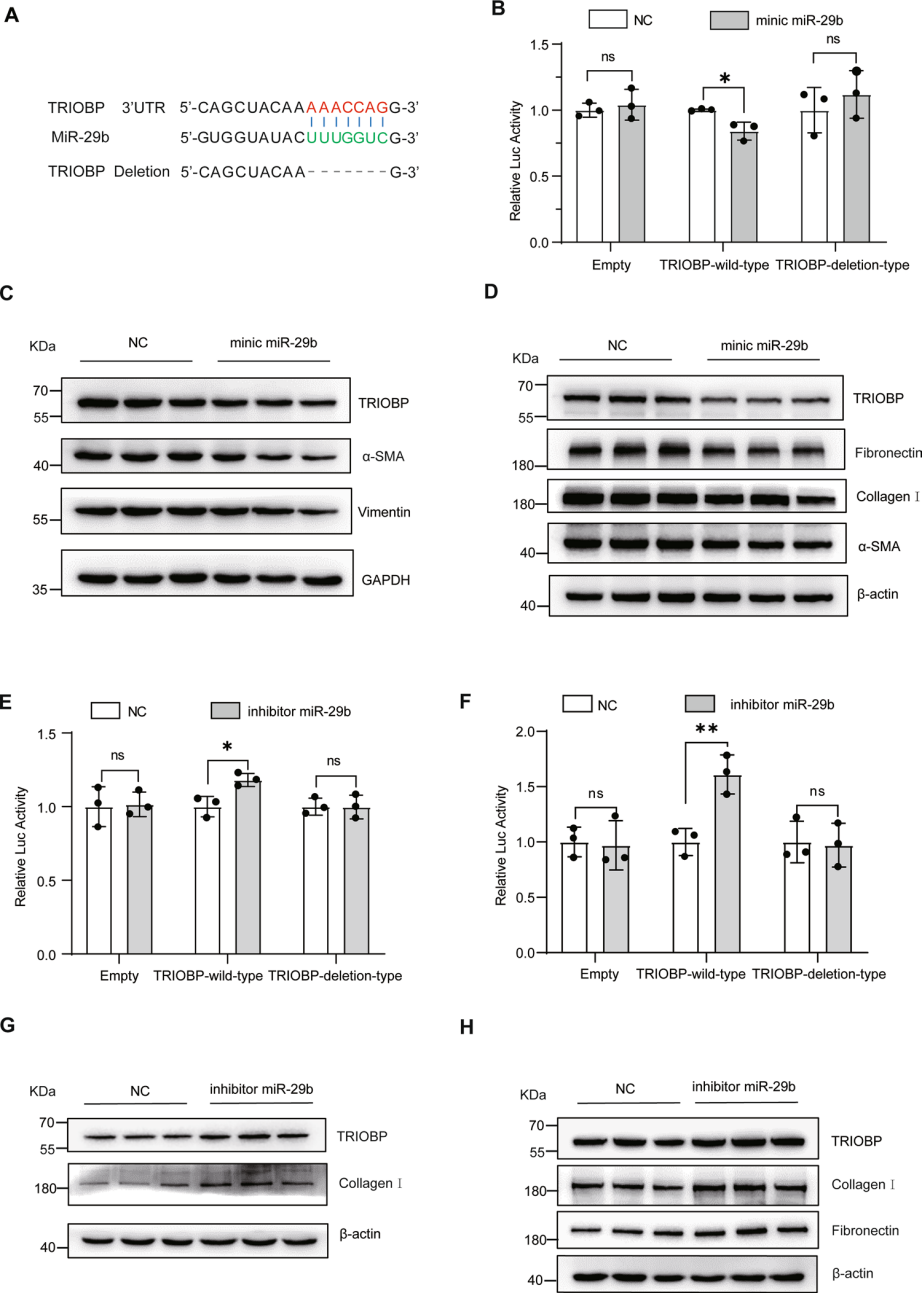
TRIOBP is one of the direct target genes of miR-29b. **A** Bioinformatics prediction analysis shows miR-29b could target the region of TRIOBP’s 3ʹUTR. **B** Luciferase assay shows mimic of miR-29b significantly decreased the luciferase activity of TRIOBP‐wild-type. *p < 0.05 when compared with the plasmid co-transfected with empty vector and TRIOBP‐wild-type; data were evaluated by two-way ANOVA with Šídák’s multiple comparisons test for pairwise comparisons (*n* = 3). **C** Western blot of TRIOBP, α-SMA, vimentin, and GAPDH in the NC and mimic miR-29b A549 cells groups. **D** Western blot of TRIOBP, α-SMA, fibronectin, collagen I, and β-actin in the NC and mimic miR-29b MRC5 cells groups. **E** The luciferase assay shows inhibitor of miR-29b significantly increased the luciferase activity of TRIOBP‐wild-type in A549 cells. *p < 0.05, data were evaluated by two-way ANOVA with Šídák’s multiple comparisons test for pairwise comparisons (*n* = 3). **F** The luciferase assay shows inhibitor of miR-29b significantly increased the luciferase activity of TRIOBP‐wild-type in MRC5 cells. *p < 0.05 when compared with the plasmid co-transfected with empty vector and TRIOBP‐wild-type; data were evaluated by two-way ANOVA with Šídák’s multiple comparisons test for pairwise comparisons (*n* = 3). **G** Western blot of TRIOBP, collagen I and β-actin in the NC and mimic miR-29b A549 cells groups. **H** Western blot of TRIOBP, collagen I, fibronectin and β-actin in the NC and mimic miR-29b MRC5 cells groups

We predicted that TRIOBP was one of the potential targets of miR-29b for its binding to the specific sequence of at the 3ʹ UTR of TRIOBP in Targetscan database (http://www.targetscan.org/vert_72/) (Fig. 1A). Using two luciferase reporter vectors, containing TRIOBP‐wild-type and TRIOBP‐deletion-type to confirm that TRIOBP was the target gene of miR-29b as shown in Fig. 1B, miR-29b reduced TRIOBP‐wild-type luciferase vector activity obviously, and in contrast, we were not able to observe a significant change in TRIOBP‐deletion-type vector activity. Then we transfected the miR-29b mimic into A549 and MRC5 cells, the protein expression of TRIOBP in A549 and MRC5 cells was reduced, expression of α-SMA and vimentin was reduced in A549 cells (Fig. 1C), expression of collagen I, fibronectin, and α-SMA was decreased in MRC5 cells (Fig. 1D). Subsequently, we transfected the inhibitor of miR-29b to A549 and MRC5 cells, the activity of the TRIOBP-wild-type luciferase vector was enhanced (Fig. 1E, F), the expression of TRIOBP and profibrotic markers, such as collagen I and fibronectin, was increased both in A549 and MRC5 cells at mRNA (Figs. S1A-B) and protein levels (Fig. 1G, H). All these data indicated that TRIOBP is one of the directly target genes of miR-29b and promote the profibrotic phenotype**.**

### Deficiency of TRIOBP increases resistance to lung fibrosis in vivo

**Fig. 2:**
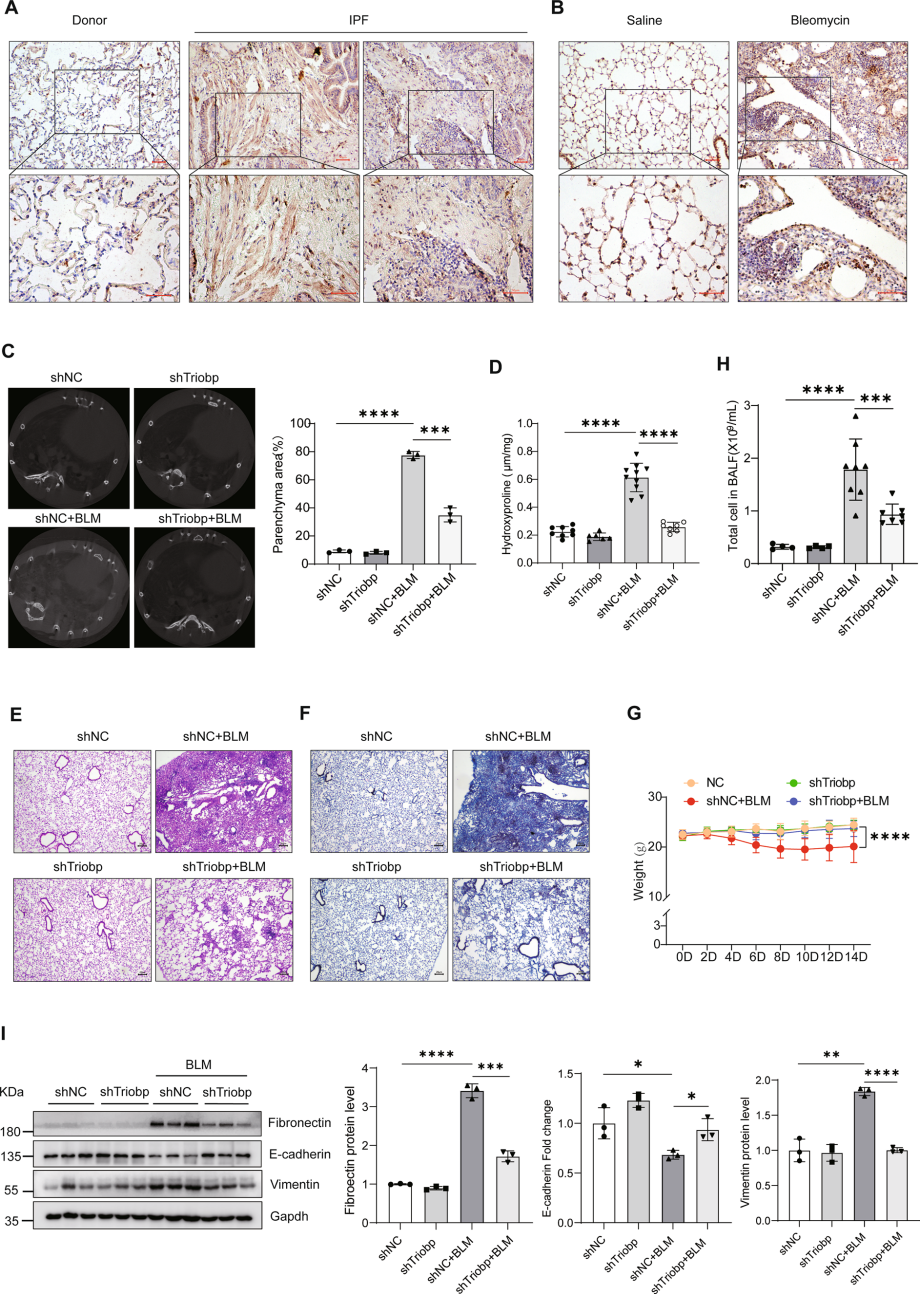
Triobp knockdown blocks the lung fibrosis in BLM-induced mice. **A** Immunohistochemical analysis indicated that TRIOBP was upregulated in IPF lung tissues. Bar = 50 μM. **B** Immunohistochemistry indicated that Triobp was upregulated in BLM-treated mice model of pulmonary fibrosis. Bar = 50 μM. **C** Micro-CT imaging system for small animals (left). Quantification of fibrotic area. Representative micro-CT images of mouse lungs (right). Micro-CT, micro-computed tomography. **D** Hydroxyproline content in mice. Data were evaluated by two-way ANOVA with Šídák’s multiple comparisons test for pairwise comparisons. Data ****p < 0.0001. **E** H&E and staining. **F** Masson staining. **G** Body weight monitoring revealed that BLM-treated mice lost substantial body mass compared with sham mice, and sh-Triobp effectively blocked this loss compared with the control group. Data were evaluated by two-way ANOVA with Šídák’s multiple comparisons test for pairwise comparisons. **H** Count of total number of cells in BALF. Data were evaluated by two-way ANOVA with Šídák’s multiple comparisons test for pairwise comparisons. Data ***p < 0.001 and ****p < 0.0001. **I** Western blot of fibronectin and vimentin, and E-cadherin expression

Increased TRIOBP expression was detected in the lung tissue of IPF patients as compared with control samples (Fig. 2A). We also observed upregulation of Triobp expression in BLM-induced mice model of pulmonary fibrosis (Fig. 2B). To evaluate the potential role of TRIOBP in lung injury and fibrosis in vivo, we generated the mice model of lung fibrosis and shRNA adenovirus targeting Triobp treatment mice model (Fig.S2). The X-ray micro-computed tomography (micro-CT) performances showed that opacifications were clearly moderated in sh-Triobp group (Fig. 2C). The lung pathology of BLM-treated mice showed extensive fibrosis with markedly massive collagen deposits, fibrotic lesions, and distorted lung architecture by H&E and Masson Trichrome staining and hydroxyproline assay (Fig. 2D–F), the sh-Triobp infected mice showed reduction collagen deposition and prevention in their weight loss (Fig. 2G), along with reduction in the inflammatory cells counts in bronchoalveolar lavage fluid (BALF) (Fig. 2H), the expression of fibronectin and vimentin was markedly reduced; however, the expression of epithelial marker E-cadherin was increased in the lungs of the sh-Triobp infection mice (Fig. 2I). These data showed that deficiency of TRIOBP decreases susceptibility to lung fibrosis in vivo.

### TRIOBP knockdown inhibited the proliferation, migration, and profibrotic gene expression of epithelial cell

**Fig. 3:**
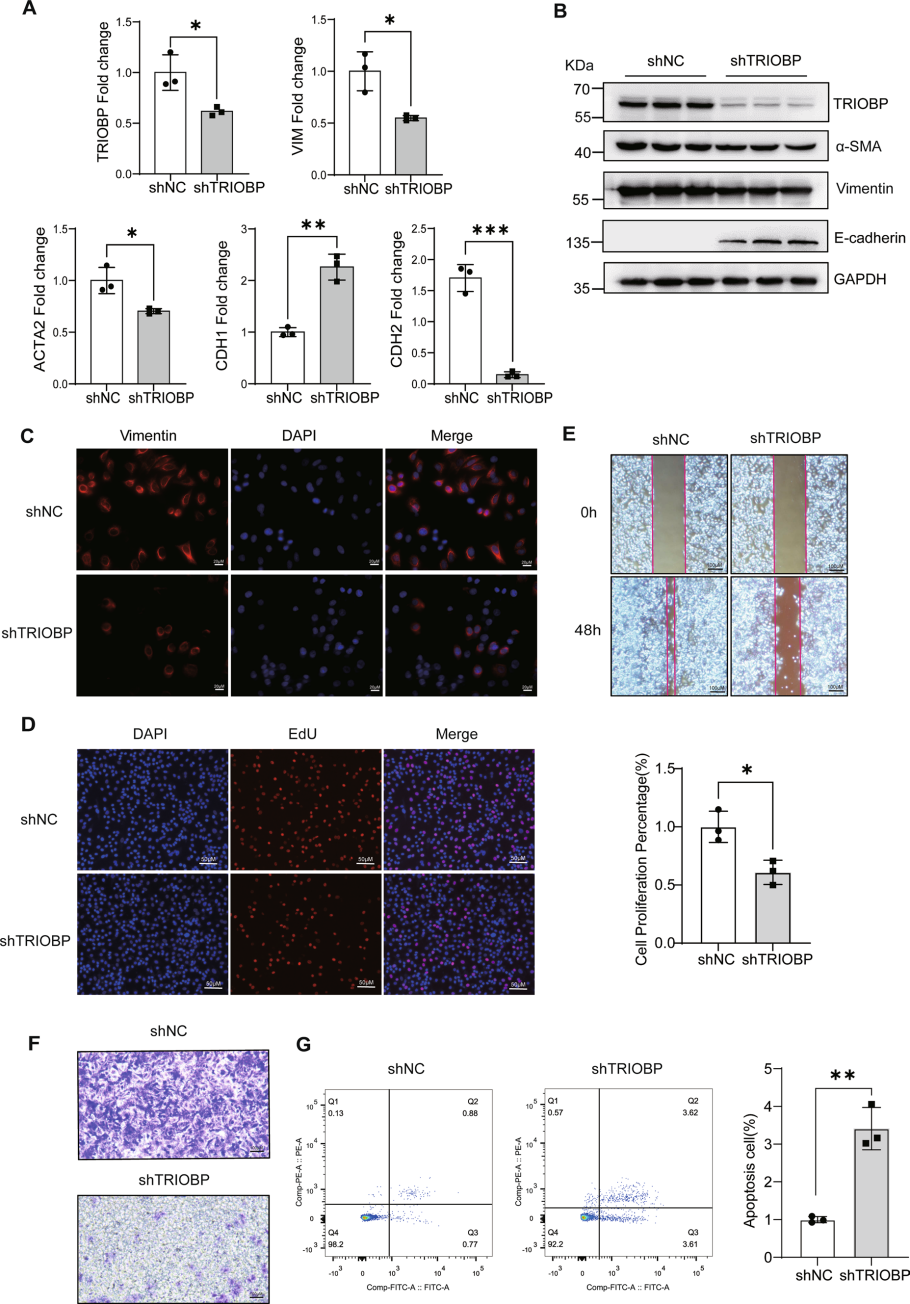
Lack of TRIOBP reduced abnormal cell proliferation, migration, EMT and promoted cell apoptosis in epithelial cells. **A** RT-qPCR of *TRIOBP* and EMT marker genes (*ACTA2*, *VIM*, *CDH1*, *CDH2*) expression (*n* = 3). *p < 0.05, **p < 0.01, and ***p < 0.001. **B** Western blot of TRIOBP, α-SMA, vimentin, E-cadherin, and β-actin in the shNC and sh-TRIOBP A549 cells groups. shNC: short hairpin control (control lentivirus); sh-TRIOBP: short hairpin TRIOBP (TRIOBP lentivirus). **C** Immunofluorescence images (using Fire LUT) of vimentin (*n* = 3). The nuclei were counterstained with DAPI. Bar = 20 μm. **D** The EdU proliferation assay was used to measure A549 cell proliferation (*n* = 3). Bar = 50 μm. *p < 0.05. **E** Wound healing assays conducted and photographed at 0 and 48 h, with quantification (*n* = 3). Bar = 100 μm. **F** Transwell migration assay of A549 cells at 36 h after TRIOBP knockdown (*n* = 3). Bar = 100 μm. **G** Cell apoptosis measured by cytometry, with quantification analysis (*n* = 3). **p < 0.01. The results were analyzed by the unpaired Student’s *t* test for comparisons between two groups with normal distribution, data are presented as mean ± SD. Data *p < 0.05, **p < 0.01, and ***p < 0.001

In vivo studies indicate that loss of TRIOBP showed robust anti-fibrotic activity, the abnormally expression of E-cadherin and vimentin in BLM-induced mice lung initiated us to assess the effect of TRIOBP in alveolar epithelium. First, TRIOBP knockdown inhibited vimentin, α-SMA, N-cadherin expression, increased E-cadherin expression in A549 cells at both mRNA and protein levels (Fig. 3A, B). Knockdown of TRIOBP also induced a decreasing of vimentin positive cells (Fig. 3C). Moreover, reduced TRIOBP expression inhibited significantly the epithelial cell proliferation and migration (Fig. 3D–F), while promoted the epithelial cell apoptosis (Fig. 3G). These findings suggested that TRIOBP elicited substantial effects in epithelium cell proliferation, migration, and EMT process, which might participate in lung tissue injury-remodeling process.

### TRIOBP silencing attenuated the activation of fibroblasts in MRC5 cells and PMLFs

**Fig. 4:**
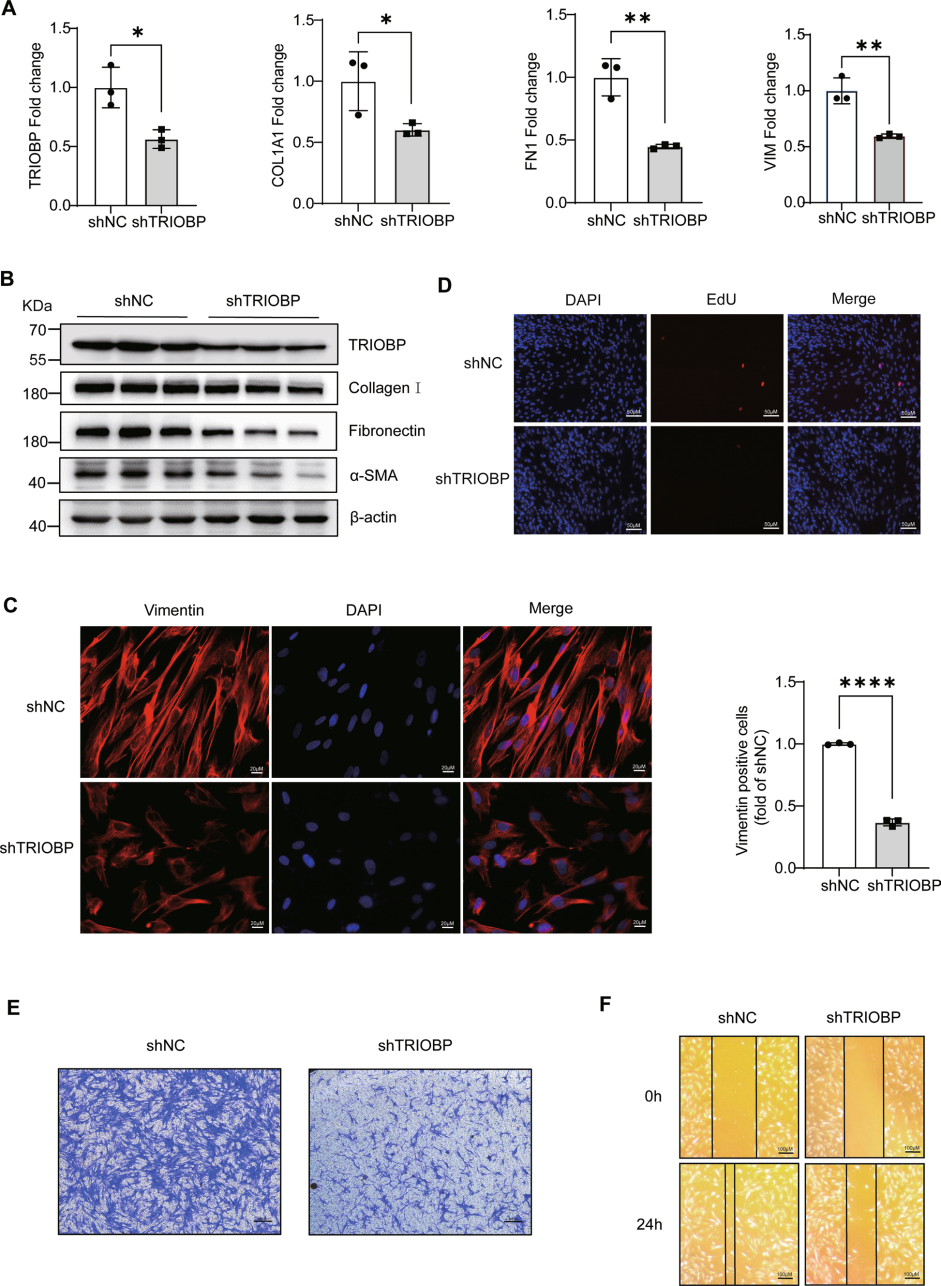
TRIOBP silencing reduced lung fibroblast activation. **A** RT-qPCR of *TRIOBP* and fibrotic marker genes (*COL1A1*, *FN1*, *VIM*) expression (*n* = 3). *p < 0.05 and **p < 0.01. **B** Western blot of TRIOBP, α-SMA, vimentin, collagen I, fibronectin, and β-actin in the shNC and sh-TRIOBP MRC5 cells groups. shNC: short hairpin control (control lentivirus); sh-TRIOBP: short hairpin TRIOBP (TRIOBP lentivirus). **C** Immunofluorescence images (using Fire LUT) of vimentin (*n* = 3). The nuclei were counterstained with DAPI. Bar = 20 μm. ****p < 0.0001. **D** The EdU proliferation assay was used to measure MRC5 cell proliferation (*n* = 3). Bar = 50 μm. **E** Transwell migration assay of MRC5 cells at 36 h after TRIOBP knockdown (*n* = 3). Bar = 100 μm. **F** Wound healing assays conducted and photographed at 0 and 24 h, with quantification (*n* = 3). Bar = 100 μm. The results in A, C were analyzed by the unpaired Student’s *t* test for comparisons between two groups with normal distribution, data are presented as mean ± SD. Data *p < 0.05, **p < 0.01, and ****p < 0.0001

Fibroblast-to-myofibroblast differentiation and myofibroblast proliferation and migration are major clinical manifestations of this disease; hence, blocking these processes is a practical treatment strategy [50]. Thus, we investigated the role of TRIOBP in lung fibroblasts using a shRNA to knockdown the TRIOBP in MRC5 cell line. TRIOBP knockdown reduced collagen I, fibronectin, and vimentin expression at both mRNA and protein levels in MRC5 cells (Fig. 4A, B). The shrinking of vimentin positive cells was obviously observed in Fig. 4C. TRIOBP knockdown also reduced the excessive proliferation and migration of MRC5 (Fig. 4D–F). We isolated PMLFs and silenced the expression of Triobp in PMLFs to further validate the function of Triobp in mice. Triobp knockdown inhibited the expression of fibronectin in PMLFs (Fig.S3). These results indicate that TRIOBP silencing attenuated the activation of lung fibroblast.

### Deficiency of TRIO in epithelial cell and fibroblast decreases susceptibility to lung fibrosis

**Fig. 5:**
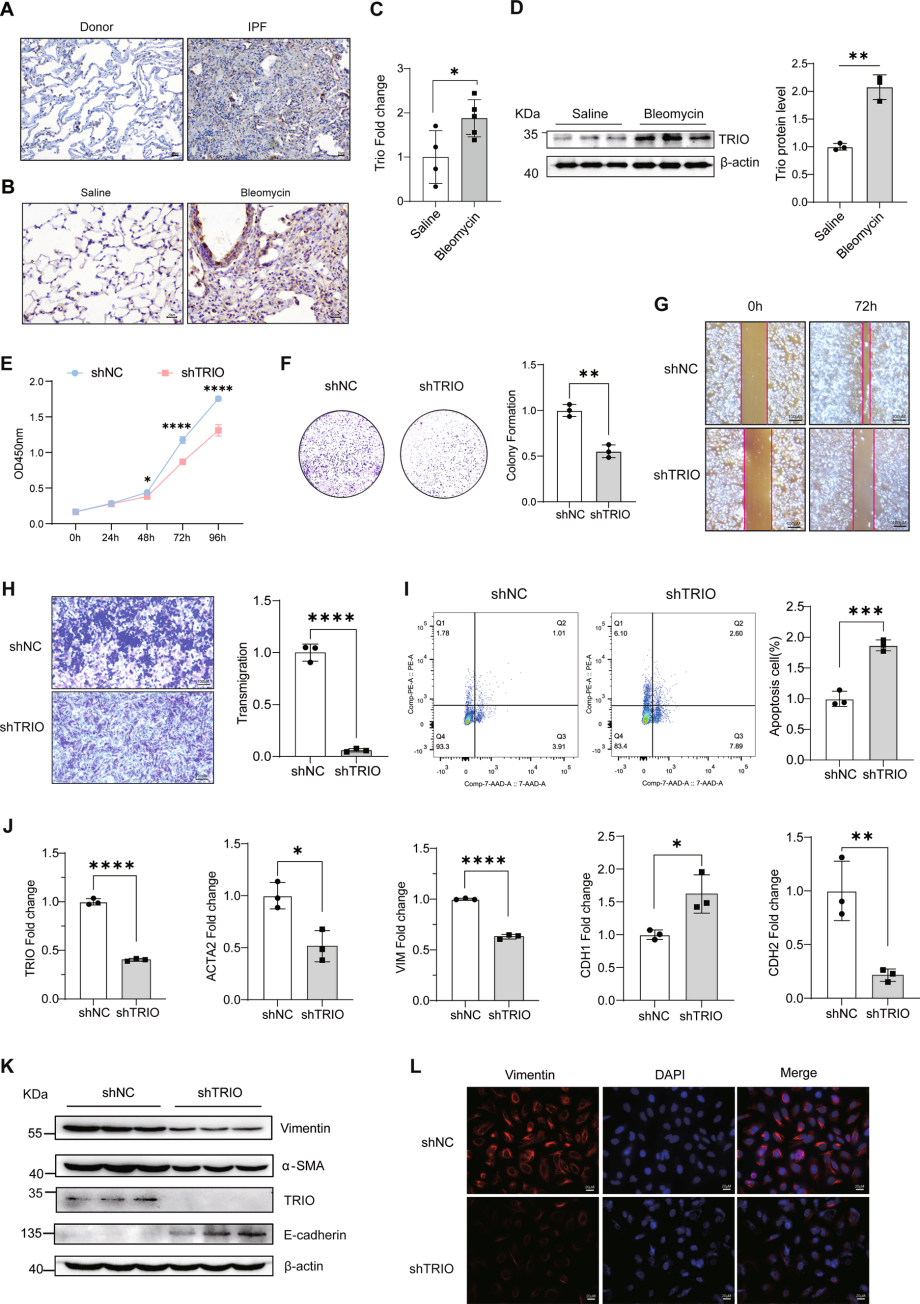
A549 cells lacking TRIO inhibits abnormal cell proliferation, migration, apoptosis, and differentiation. **A** Immunohistochemical analysis indicated that TRIO was upregulated in IPF lung tissues. Bar = 20 μM. **B** Immunohistochemistry indicated that TRIO was upregulated in BLM-treated mouse lung. Bar = 20 μM. **C** RT-qPCR tested the mRNA level of *TRIO* in BLM-treated mouse lung. The results were analyzed by the unpaired Student’s *t* test for comparisons between two groups with normal distribution, data are presented as mean ± SD. Data *p < 0.05. **D** Western blot tested the protein level of TRIO in BLM-treated mouse lung tissues. **E** CCK8 assay at 0, 24, 48, 72, 96 h (*n* = 5). *p < 0.05 and ****p < 0.0001. **F** The effect of TRIO knockdown on the colony formation of A549 cells (*n* = 3). shNC: short hairpin control (control lentivirus); sh-TRIO: short hairpin TRIO (TRIO lentivirus). **p < 0.01. **G** Wound healing assays conducted and photographed at 0 and 72 h, with quantification (*n* = 3). Bar = 100 μm. **H** Transwell migration assay of A549 cells at 36 h after TRIO knockdown (*n* = 3). Bar = 100 μm. ****p < 0.0001.** I** Cell apoptosis measured by cytometry, with quantification analysis (*n* = 3). ***p < 0.001.** J** RT-qPCR of *TRIO* and EMT marker genes (*ACTA2*, *VIM*, *CDH1*, *CDH2*) expression (*n* = 3). *p < 0.05, **p < 0.01 and ****p < 0.0001. **K** Western blot of TRIO, α-SMA, vimentin, E-cadherin and β-actin in the shNC and sh-TRIO A549 cells groups.** L** Immunofluorescence images (using Fire LUT) of vimentin (*n* = 3). The nuclei were counterstained with DAPI. Bar = 20 μm. Data in A were evaluated by two-way ANOVA with Šídák’s multiple comparisons test for pairwise comparisons. The results were analyzed by the unpaired Student’s *t* test for comparisons between two groups with normal distribution, data are presented as mean ± SD. Data *p < 0.05, **p < 0.01, ***p < 0.001, and ****p < 0.0001

**Fig. 6:**
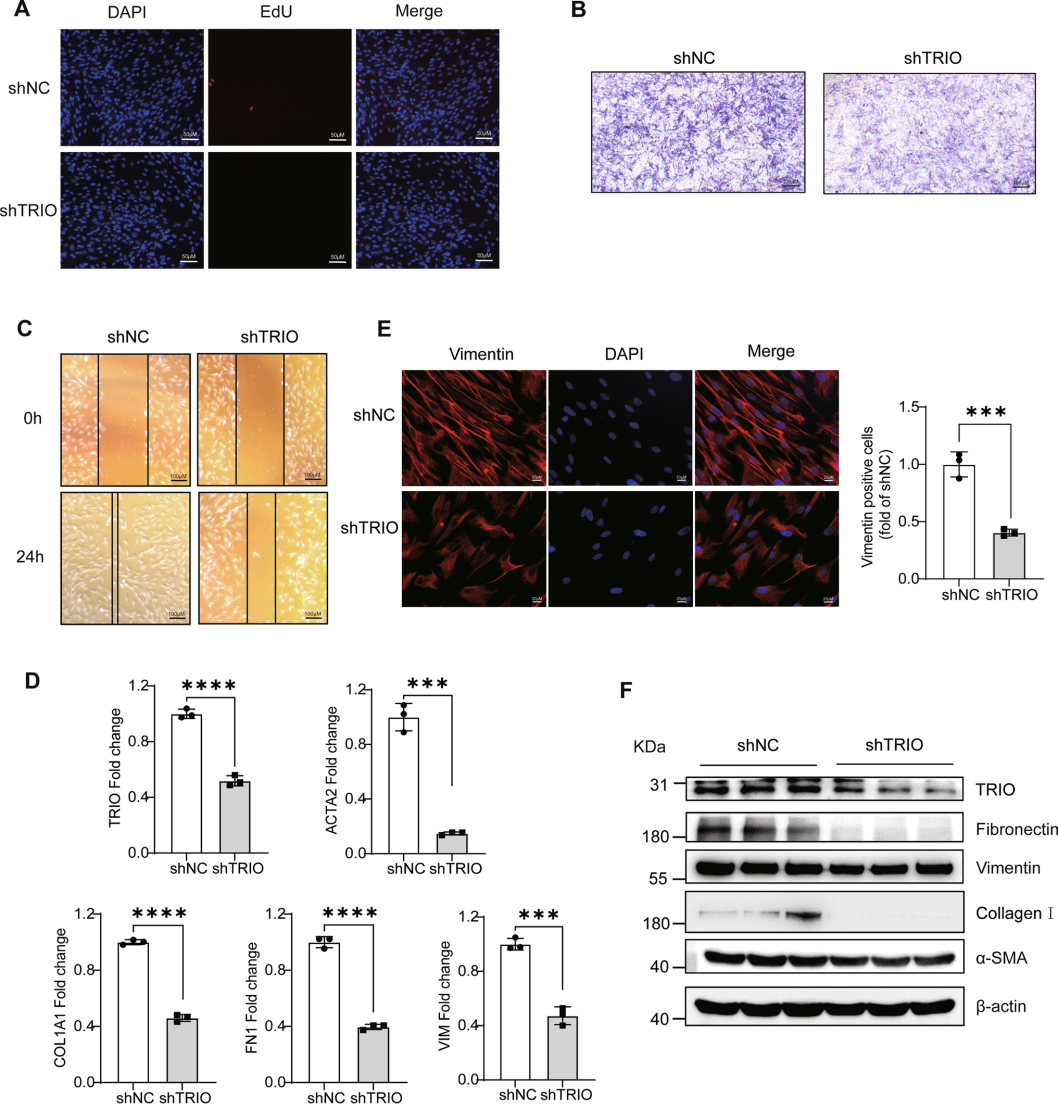
TRIO silencing decreases the fibroblast proliferation, migration, and activation. **A** The EdU proliferation assay was used to measure MRC5 cell proliferation (*n* = 3). Bar = 50 μm. **B** Transwell migration assay of MRC5 cells at 36 h after TRIO knockdown (*n* = 3). Bar = 100 μm. **C** Wound healing assays conducted and photographed at 0 and 24 h (*n* = 3). Bar = 100 μm. **D** RT-qPCR of *TRIO* and fibrotic marker genes (*ACTA2*, *COL1A1*, *FN1*, *VIM*) expression (*n* = 3). ***p < 0.001 and ****p < 0.0001. **E** Immunofluorescence images (using Fire LUT) of vimentin (*n* = 3). The nuclei were counterstained with DAPI. Bar = 20 μm. ***p < 0.001. **F** Western blot of TRIO, α-SMA, vimentin, collagen I, fibronectin, and β-actin in the shNC and sh-TRIO MRC5 cells groups. The results were analyzed by the unpaired Student’s *t* test for comparisons between two groups with normal distribution, data are presented as mean ± SD. Data ***p < 0.001 and ****p < 0.0001

As known, TRIOBP interacts with TRIO, which is controlling actin cytoskeleton organization, cell motility, and cell growth [51]. We found that expression of TRIO was higher in lungs of patients with IPF compared with healthy donors and in BLM-induced lung of mice (Fig. 5A–D). The TRIO and TRIOBP expression and distribution were shown in the UMAP by analyzing the single-cell RNA-seq data from Human Lung Cell Atlas (Fig.S4A) and mouse single-cell data from our previous work (Fig.S4B). In A549 and MRC5 cells, the expression of TRIOBP was positively correlated with the expression of TRIO (Fig.S4C–F). To determine the role of TRIO in epithelial injury and fibrosis, we generated the TRIO knockdown A549 cell and MRC5 cell. TRIO knockdown inhibited cell proliferation and migration significantly (Figs. 5E–H, 6A–C) in A549 and MRC5 cell, resulted in apoptosis in A549 (Fig. 5I).

A549 cells lacking TRIO resulted in significant lower transcript level of *ACTA2*, *VIM*, *CDH2* and lower protein level of α-SMA accompanied by higher transcript level of *CDH1* and E-cadherin (Fig. 5J–L). Similarly, Trio knockdown inhibited the expression of vimentin and α-SMA, as well as reduced the proliferation of mouse epithelium MLE-12 cells (Fig.S5). In MRC5 cells, TRIO knockdown decreased the transcript level of *ACTA2*, *VIM*, *COL1A1*, *FN1* (Fig. 6D), and the protein expression of α-SMA, vimentin, collagen I, and fibronectin (Fig. 6E, F). Therefore, we concluded that deficiency of TRIO in epithelial cell and fibroblast decreases susceptibility to lung fibrosis.

### TRIOBP interacting with TRIO promoted abnormal epithelial–mesenchymal crosstalk

**Fig. 7:**
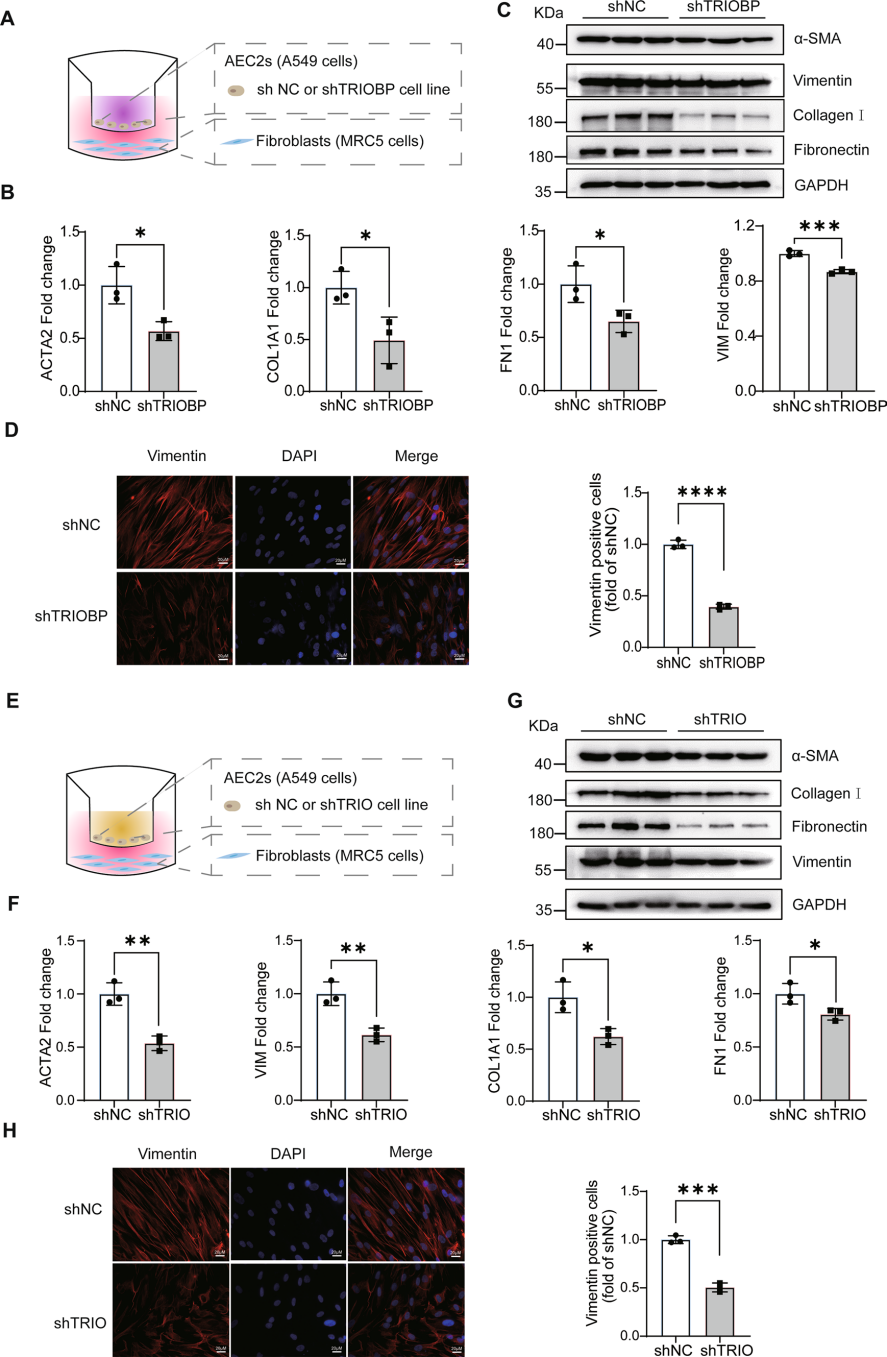
TRIO and TRIOBP knockdown attenuated the abnormal epithelial–mesenchymal crosstalk. **A** The model of A549 cells co-cultured with human lung fibroblasts. A549 shNC and sh-TRIOBP cell lines, as well as normal human lung fibroblast (MRC5 cells) were used in this system. The MRC5 cells were used to experiment after cultured for 48 h. **B** RT-qPCR of co-cultured MRC5 cells’ (**A**) *ACTA2*, *COL1A1*, *FN1*, and *VIM* expression. *p < 0.05 and ***p < 0.001. **C** Western blot of co-cultured MRC5 cells’ (**A**) α-SMA, collagen I, fibronectin, vimentin, and GAPDH expression (*n* = 3). **D** Above **A**’s co-cultured system, immunofluorescence images (using Fire LUT) of vimentin (*n* = 3). The nuclei were counterstained with DAPI. Bar = 20 μm. ****p < 0.0001. **E** The model of A549 cells co-cultured with human lung fibroblasts. A549 shNC and sh-TRIO cell lines, as well as normal human lung fibroblast (MRC5 cells) were used in this system. The MRC5 cells were used to experiment after cultured for 48 h. **F** RT-qPCR of co-cultured MRC5 cells’ (**E**) *ACTA2*, *COL1A1*, *FN1* and *VIM* expression. *p < 0.05 and **p < 0.01. **G** Western blot of co-cultured MRC5 cells’ (**E**) α-SMA, collagen I, fibronectin, vimentin, and GAPDH expression (*n* = 3). **H** Above **E**’s co-cultured system, immunofluorescence images (using Fire LUT) of vimentin (*n* = 3). The nuclei were counterstained with DAPI. Bar = 20 μm. ***p < 0.001. The results were analyzed by the unpaired Student’s *t* test for comparisons between two groups with normal distribution, data are presented as mean ± SD. Data *p < 0.05, **p < 0.01, ***p < 0.001, and ****p < 0.0001

As previously proved that TRIOBP silencing inhibits the proliferation and migration of epithelial cell, TRIO knockdown reduces the proliferation of A549 and MLE-12 cells, while TRIOBP was positively correlated with the expression of TRIO in A549 and MRC5 cells; therefore, we proposed that the TRIOBP might cause abnormal AECIIs injury and repair, and this abnormal activation might cause dysregulated crosstalk between the epithelium and mesenchymal cells as well as an accumulation of myofibroblasts. As shown in Fig. 7A, TRIOBP knockdown of A549 was co-culture with fibroblast to investigate the expression of α-SMA, vimentin, collagen I, and fibronectin in MRC5 cells at both the mRNA and protein levels, all of them were inhibited compared to the controls (Fig. 7B–D), the same results were observed when TRIO knockdown of A549 was co-cultured with fibroblast (Fig. 7E–H). These results support our in vitro findings that TRIOBP promoted abnormal epithelial–mesenchymal crosstalk by regulating TRIO expression.

### TRIOBP/TRIO modulates the nucleocytoplasmic translocation of β-catenin

**Fig. 8:**
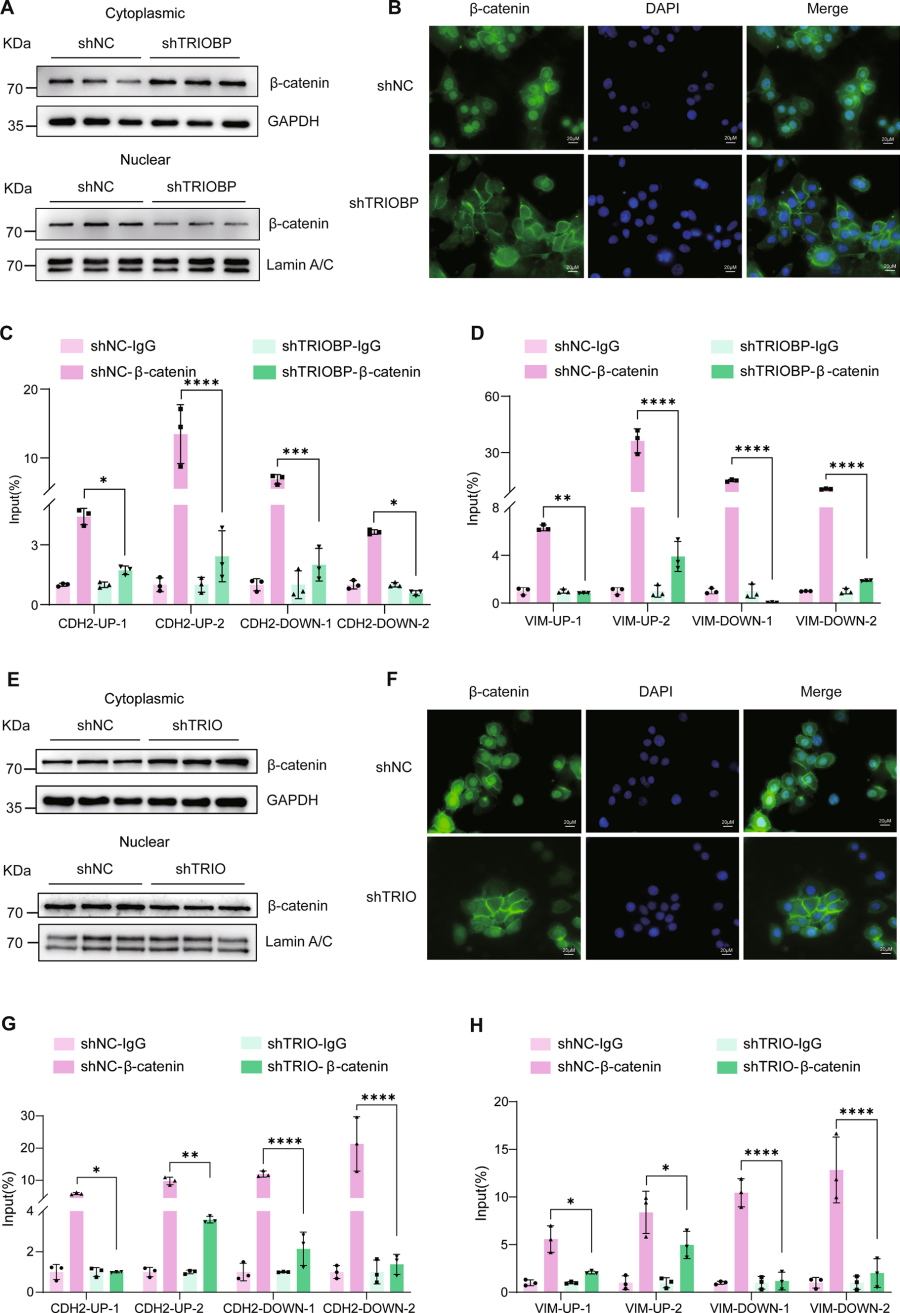
TRIO knockdown triggers the nucleocytoplasmic translocation of β-catenin. **A** Western blot of β-catenin in the nucleus and cytoplasm in the shNC and sh-TRIOBP groups in A549 cells. **B** Immunofluorescence images (using Fire LUT) of β-catenin. The nuclei were counterstained with DAPI. Bar = 20 μm. **C** ChIP assay of β-catenin binding to the *CDH2* promoter in A549 cells from shNC and sh-TRIOBP group. **D** ChIP assay of β-catenin binding to the *VIM* promoter in A549 cells from shNC and sh-TRIOBP group. **E** Western blot of β-catenin in the nucleus and cytoplasm in the shNC and sh-TRIO groups in A549 cells. **F** Immunofluorescence staining of β-catenin. The nuclei were counterstained with DAPI. Bar = 20 μm. **G** ChIP assay of β-catenin binding to the *CDH2* promoter in A549 cells from shNC and sh-TRIO group. **H** ChIP assay of β-catenin binding to the *VIM* promoter in A549 cells from shNC and sh-TRIO group. Data were evaluated by two-way ANOVA with Šídák’s multiple comparisons test for pairwise comparisons. Data *p < 0.05, **p < 0.01, ***p < 0.001, and ****p < 0.0001

Transcription factor β-catenin is involved in alveolar epithelial–mesenchymal transition during pulmonary fibrosis, we hypnotized that TRIOBP interacts with TRIO modulating fibrosis through β-catenin signal pathway. Analysis of the nuclear and cytoplasmic protein levels of β-catenin demonstrated that knockdown of TRIOBP increased the level of β-catenin in the cytoplasmic, while decreased it in the nucleus (Fig. 8A). Immunofluorescence staining also revealed that knockdown of TRIOBP inhibited the localization of β-catenin in nucleus (Fig. 8B). To further examine whether TRIOBP affects the binding of β-catenin as a transcription factor to the promoter regions of *VIM* and *CDH2*, ChIP-qPCR results revealed that β-catenin exhibited significant binding to the promoter regions of *CDH2* and *VIM* in the control group; however, TRIOBP knockdown markedly reduced the binding ability of β-catenin to the promoter regions of *CDH2* and *VIM* in A549 cells (Fig. 8C, D). Next, we investigated whether TRIO knockdown also changes the nucleocytoplasmic translocation of β-catenin. Western blot and immunofluorescence staining showed that TRIO silencing also deregulated the disposition of β-catenin in nucleus (Fig. 8E, F). Moreover, similarly, ChIP-qPCR results revealed that TRIO knockdown blocked β-catenin bounds to promoter regions of *VIM* and *CDH2* (Fig. 8G, H). These data suggest that TRIOBP and TRIO are critical for the binding of β-catenin to profibrotic genes and affected epithelial–mesenchymal crosstalk.

Taken together, these findings imply that TRIOBP is one of target genes of miR-29b. TRIOBP interacts with TRIO to promote abnormal epithelial–mesenchymal crosstalk and downstream β-catenin signaling in IPF. The miR-29b–TRIOBP–TRIO–β-catenin axis might be a key anti-fibrotic axis and provide a promising treatment strategy for lung fibrosis.

## Discussion

Our previous studies demonstrated that miR-29 mimicry inhibited pulmonary fibrosis in BLM-treated mice, rats, and non-human primates [28, 29], but little was known about the mechanism underlined in the models and patients. Here, we showed that miR-29b could bind to TRIOBP which interact with TRIO, the miR-29–TRIOBP–TRIO axis that modulates activation of the β-catenin signaling pathway in IPF. Our findings exhibit that TRIOBP is one of the target genes and discovers a novel mechanism that miR-29b blocks pulmonary fibrosis by regulation of TRIOBP and TRIO.

MiRNAs have been shown to predict IPF mortality and facilitate early diagnosis. miR-185, miR-210, miR-302c, miR-376c, and miR-423-5p were increased in IPF lung tissue of rapid progressors, the reduced miR-29 expression in peripheral blood was associated with increased mortality of patients with IPF [52]. MiRNAs act as negative regulators of gene expression by inhibiting the translation or promoting the degradation of target mRNAs, individual miRNAs often regulate the expression of multiple target genes with related functions, a change in the expression of a single miRNA, or a family sharing the same targets, can influence an entire gene network and thereby modify complex disease phenotypes in principle [53]. In the context of their targets, miRNAs are deeply involved in regulating processes implicated in IPF such as lung differentiation and development, regulation of ECM deposition and TGF β signaling [54]. The miR-29 family is probably the most relevant to fibrosis. The family consists of miR-29a, -29b and -29c, which are expressed as 2 bicistronic clusters (miR-29a/-29b-1 and miR-29b-2/-29c), and are largely homologous in sequence [27]. Among the predicted and proven targets of the miR-29 family are multiple ECM proteins and profibrotic molecules, herein we discovered that TRIOBP is one of the target genes of miR-29b, miR-29b inhibited the expression of TRIOBP at the mRNA and protein levels in A549 and MRC5 cells. Silencing TRIOBP reduced the ECM disposition and inhibited the EMT process in vivo*.* TRIOBP knockdown inhibited the abnormal activation of AECIIs and fibroblast. The most accepted in pathogenesis of IPF is the epithelial-driven process triggered by AECIIs injury which leads to altered crosstalk with immune cells, fibroblasts, myofibroblast activation, and ECM accumulation [1, 50, 55, 56]. Next, we explored the molecular mechanism of TRIOBP in pulmonary fibrosis. As a binding partner of TRIO [51], TRIOBP positively regulated TRIO at mRNA and protein levels in this study. TRIO knockdown inhibited profibrotic genes expression, while increased the expression of E-cadherin in AECIIs. TRIO knockdown inhibited the fibroblast differentiation to myofibroblast and ECM disposition in vitro. TRIOBP positively regulated TRIO expression and TRIO plays a similar role of TRIOBP in AECIIs and lung fibroblast. Activated alveolar epithelial cells could release a variety of cytokines and profibrogenic growth factors, which resulting in aberrant epithelial–mesenchymal crosstalk and myofibroblast activation, with deposition and remodeling of the ECM. Interestingly, we found that TRIOBP and TRIO knockdown in AECIIs both inhibited the activation of lung fibroblast. Thus, we think that the expression of TRIOBP in AECIIs affects lung fibroblast through regulating TRIO expression. To further explore the mechanism of TRIO regulating epithelial–mesenchymal crosstalk, we found that TRIO silencing decreased transportation of β-catenin to the nucleus and TRIO is critical for the binding of β-catenin to profibrotic genes chromatin in A549 cells. These results pinpoint that TRIO affects the translocation of β-catenin in nuclear, thereby regulating the activation of lung fibroblasts and suggest that miR-29b blocks EMT and lung fibroblast activation in IPF by targeting TRIOBP regulation of TRIO. Further TRIO is critical for the binding of β-catenin to chromatin for profibrotic genes expression and TRIO affected epithelial–mesenchymal crosstalk through regulating AECIIs’ β-catenin transfer to nuclear.

In conclusion, the present work revealed the role and mechanism of the miR-29b–TRIOBP–TRIO–β-catenin axis in lung fibrosis and provided a better understanding for IPF. The results from in vitro and in vivo experiments indicated that increasing with miR-29b or interfering with TRIOBP expression may be effective strategies for the prevention and treatment of lung fibrosis.

### Supplementary Information

Below is the link to the electronic supplementary material.

Supplementary file1 Supplemental figure 1 (A) RT-qPCR test showed that inhibitor miR-29b increased the mRNA level of TRIOBP, ACTA2, VIM, COL1A1 and CDH2 in A549 cells. (B) RT-qPCR test showed that inhibitor miR-29b increased the mRNA level of TRIOBP, ACTA2, VIM, COL1A1 and FN1 in MRC5 cells. The results were analyzed by the unpaired Student’s t-test for comparisons between two groups with normal distribution, data are presented as mean±SD. Data *p<0.05, **p<0.01 and ***p<0.001 (TIF 60334 KB)

Supplementary file2 Supplemental figure 2 (A) The model of AVVs and BLM-induced mouse model. (B-C) RT-qPCR (n=5) and western blot (n=3) test the knockdown efficiency of sh-TRIOBP-shRNA. (D) IHC staining showed TRIOBP increased in fibrotic lung. Date were analyzed by the unpaired Student’s t-test for comparisons between two groups with normal distribution, data are presented as mean±SD. Data **p<0.01 and ***p<0.001 (TIF 66293 KB)

Supplementary file3 Supplemental figure 3 Western blot test showed the expression of Triobp, fibronectin and Gapdh in PMLFs (TIF 6615 KB)

Supplementary file4 Supplemental figure 4 (A) Lung single cell RNA-seq data analysis of human, specifically focusing on the cell types present in the human lung. (B) Analysis of mouse single-cell data. (C) RT-qPCR test showed that TRIOBP silencing inhibited the mRNA level of TRIO in A549 cells. (D) Western blot of TRIO and GAPDH expression. TRIOBP knockdown inhibited the expression of TRIO in A549 cells. (E) RT-qPCR test showed that TRIOBP silencing inhibited the mRNA level of TRIO in MRC5 cells. (F) Western blot of TRIO and β-actin expression. TRIOBP knockdown inhibited the expression of TRIO in MRC5 cells. Date were analyzed by the unpaired Student’s t-test for comparisons between two groups with normal distribution, data are presented as mean±SD. Data *p<0.05, **p<0.01, ***p<0.001 and ****p<0.0001 (TIF 119535 KB)

Supplementary file5 Supplemental figure 5 (A) Western blot of Trio, E-cadherin, vimentin, α-SMA and β-actin expression (n=3). Trio knockdown inhibited the EMT process of MLE-12 cells. (B) The effect of Trio knockdown on the colony formation of MLE-12 cells. shNC: control lentivirus; shTrio: Trio lentivirus (TIF 24640 KB)

Supplementary file6 (DOCX 20 KB)

## Data Availability

All data are contained within the manuscript.

## Abbreviations

AECIIs: Alveolar epithelial type II cells
IPF: Idiopathic pulmonary fibrosis
TGF-β: Transforming growth factor-β
CM: Conditioned media
ECM: Extracellular matrix
EMT: Epithelial–mesenchymal transition
α-SMA: α-Smooth muscle actin
PI: Propidium iodide
TRIO: Trio rho guanine nucleotide exchange factor
TRIOBP: TRIO And F-Actin Binding Protein
IGF-1: Insulin-like-growth factor-1
CTGF: Connective tissue growth factor
miR-29b: MicroRNA-29b
ID: Intellectual disability
ASD: Autism spectrum disorders
OS: Osteosarcoma
MiRNAs: MicroRNAs
IHC: Immunohistochemistry
FBS: Fetal bovine serum
