# Supplementary figures and images for: TRIOBP modulates β-catenin signaling by regulation of miR-29b in idiopathic pulmonary fibrosis
Source: Cell Mol Life Sci. 2023 Dec 29;81(1):13. doi: 10.1007/s00018-023-05080-4 (PMC10756874; doi:10.1007/s00018-023-05080-4)

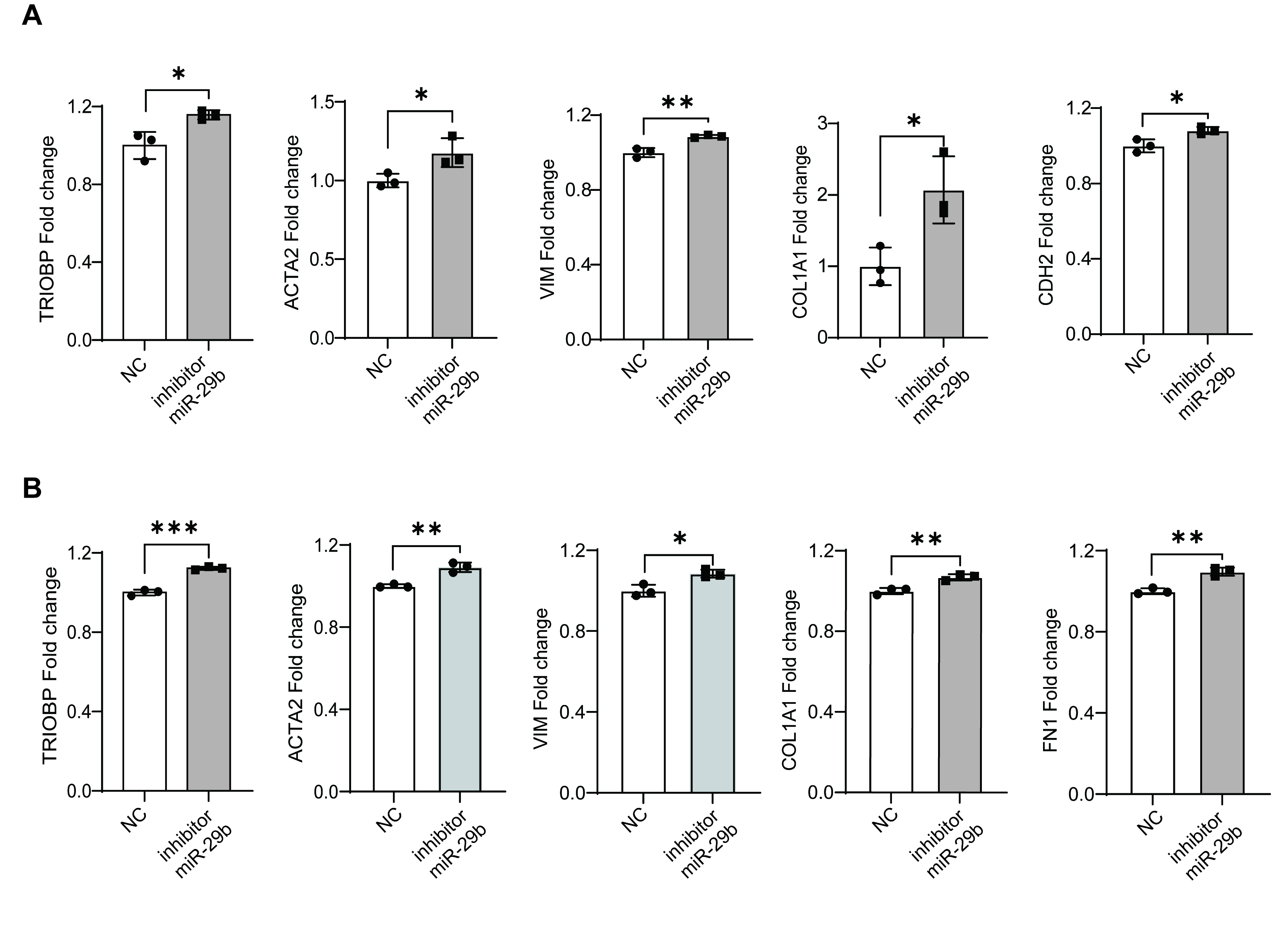

Supplement: Supplementary file 1 — Supplementary file1 Supplemental figure 1 (A) RT-qPCR test showed that inhibitor miR-29b increased the mRNA level of TRIOBP, ACTA2, VIM, COL1A1 and CDH2 in A549 cells. (B) RT-qPCR test showed that inhibitor miR-29b increased the mRNA level of TRIOBP, ACTA2, VIM, COL1A1 and FN1 in MRC5 cells. The results were analyzed by the unpaired Student’s t-test for comparisons between two groups with normal distribution, data are presented as mean±SD. Data *p<0.05, **p<0.01 and ***p<0.001 (TIF 60334 KB) [file 18_2023_5080_MOESM1_ESM.tif]

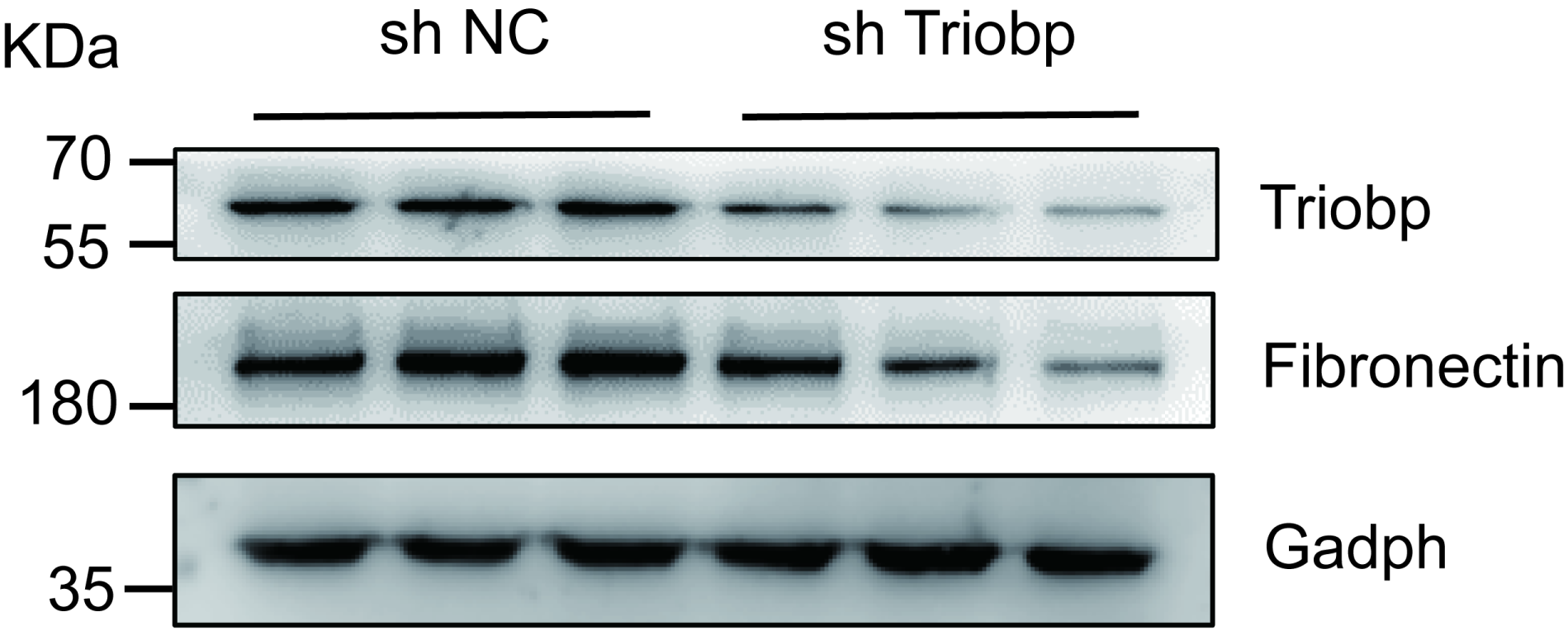

Supplement: Supplementary file 3 — Supplementary file3 Supplemental figure 3 Western blot test showed the expression of Triobp, fibronectin and Gapdh in PMLFs (TIF 6615 KB) [file 18_2023_5080_MOESM3_ESM.tif]

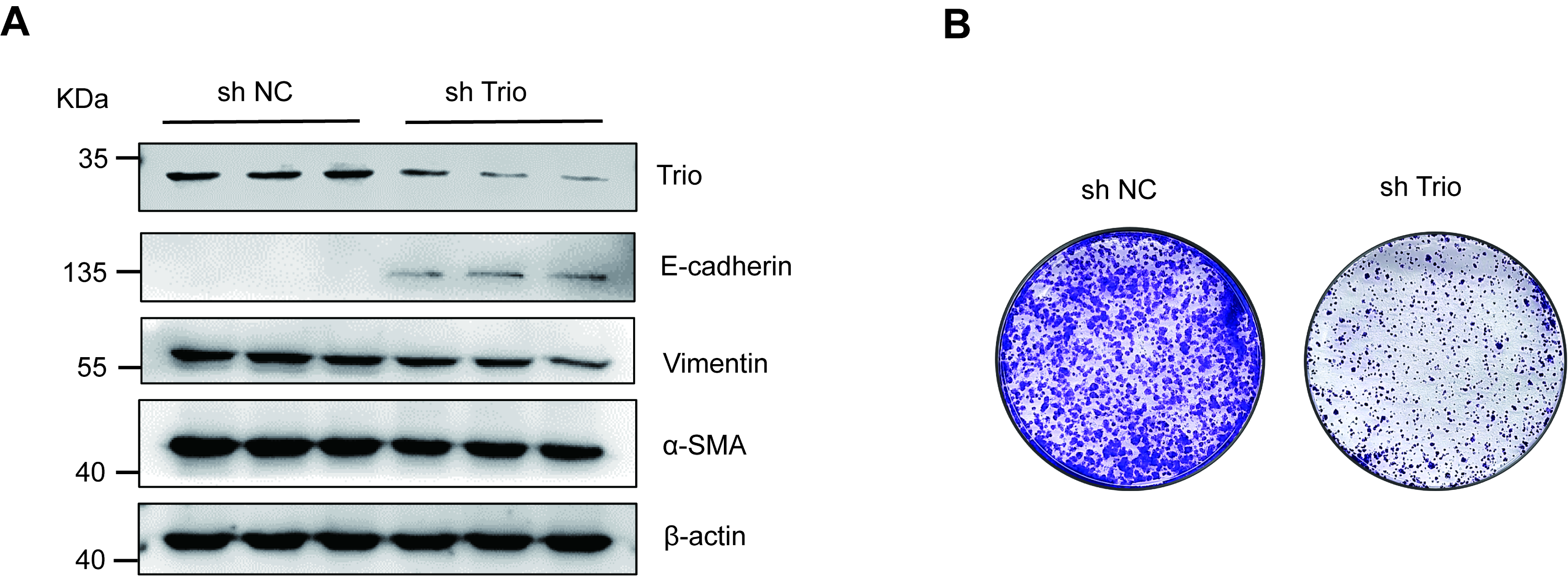

Supplement: Supplementary file 5 — Supplementary file5 Supplemental figure 5 (A) Western blot of Trio, E-cadherin, vimentin, α-SMA and β-actin expression (n=3). Trio knockdown inhibited the EMT process of MLE-12 cells. (B) The effect of Trio knockdown on the colony formation of MLE-12 cells. shNC: control lentivirus; shTrio: Trio lentivirus (TIF 24640 KB) [file 18_2023_5080_MOESM5_ESM.tif]
