# Supplementary material for: TRIOBP modulates β-catenin signaling by regulation of miR-29b in idiopathic pulmonary fibrosis
Source: Cell Mol Life Sci. 2023 Dec 29;81(1):13. doi: 10.1007/s00018-023-05080-4 (PMC10756874; doi:10.1007/s00018-023-05080-4)
Supplement: Supplementary file 6 — Supplementary file6 (DOCX 20 KB) [file 18_2023_5080_MOESM6_ESM.docx]

Supporting information

Table S1 Clinical information of all patients’ samples was used for immunohistochemistry in Fig. S3A.

Sample. Gender Age (year) Smoking status Diagnosis

IPF F 67 former Idiopathic pulmonary fibrosis

CTRL F 56 former NAT lung cancer without fibrosis

Note: F=Female. NAT= adjacent normal tissue
